# Barley Leaf Ameliorates *Citrobacter-rodentium*-Induced Colitis through Arginine Enrichment

**DOI:** 10.3390/nu15081890

**Published:** 2023-04-14

**Authors:** Yu Feng, Daotong Li, Chen Ma, Xiaosong Hu, Fang Chen

**Affiliations:** College of Food Science and Nutritional Engineering, National Engineering Research Center for Fruit and Vegetable Processing, Key Laboratory of Fruit and Vegetables Processing Ministry of Agriculture, Engineering Research Centre for Engineering Vegetables Processing, Ministry of Education, China Agricultural University, Beijing 100083, China; fengyu9459@163.com (Y.F.); lidaotong@bjmu.edu.cn (D.L.); machen21@sina.com (C.M.); huxiaos@263.net (X.H.)

**Keywords:** IBD, BL, *Citrobacter rodentium*, arginine, gut microbiota, *Akkermansia*, dose-dependent

## Abstract

Inflammatory bowel disease (IBD) has become a global public health challenge. Our previous study showed that barley leaf (BL) significantly reduces *Citrobacter-rodentium* (CR)-induced colitis, but its mechanism remains elusive. Thus, in this study, we used non-targeted metabolomics techniques to search for potentially effective metabolites. Our results demonstrated that dietary supplementation with BL significantly enriched arginine and that arginine intervention significantly ameliorated CR-induced colitis symptoms such as reduced body weight, shortened colon, wrinkled cecum, and swollen colon wall in mice; in addition, arginine intervention dramatically ameliorated CR-induced histopathological damage to the colon. The gut microbial diversity analysis showed that arginine intervention significantly decreased the relative abundance of CR and significantly increased the relative abundance of *Akkermansia*, *Blautia*, *Enterorhabdus*, and *Lachnospiraceae*, which modified the CR-induced intestinal flora disorder. Notably, arginine showed a dose-dependent effect on the improvement of colitis caused by CR.

## 1. Introduction

Inflammatory bowel disease (IBD) is a group of chronic non-specific gastrointestinal diseases that is mainly comprised of Crohn’s disease, ulcerative colitis, and unclassified IBD [1,2]. Its clinical manifestations are abdominal pain, diarrhea, bloody stool, and weight loss. IBD not only seriously affects the quality of life of patients, but also imposes a heavy economic burden on society, and has become a global public health threat [3]. However, the current traditional treatment modalities have limited efficacy and significant side effects, so there is an urgent need to develop new treatments for IBD.

IBD is a complex multifactorial disease; although its exact etiology is unclear, it has been found that gut microbiota plays an essential role in the pathogenesis of IBD [4]. The gut microbiota performs an indispensable role in maintaining the wellness of the host, while the host’s diet and living environment also affect the gut microbiota’s structure [5]. Consequently, dietary regulation of intestinal flora to improve IBD has received increasing attention. For example, barley leaf insoluble dietary fiber alleviates experimental colitis by modulating gut microbiota [6]; Tatsuya et al. demonstrated that oyster extract derived from *Crassostrea gigas* improved the intestinal flora structure, increased short-chain fatty acids (SCFAs) content, and thus improved the DSS-induced colitis [7]; and Sun et al. revealed that an excessive use of litchi causes intestinal flora dysbacteriosis, elevated serum levels of TNF-α and LPS, and destruction of the intestinal mucosal layer, causing intestinal inflammation [8].

Additionally, the gut microbiota affects host physiological functions through small molecules resulting from host and microbiota metabolism. These molecules, including SCFAs, bile acids, amino acids, polyamines, etc., are heavily influenced by dietary nutrients [9,10,11]. Arginine (Arg) is a semi-essential amino acid that is synthesized via the intestinal–renal axis and plays an important role in maintaining host health. Numerous studies have shown that Arg administration to genetically obese rats or diet-induced obese rats dramatically increases insulin sensitivity and decreases blood glucose, implying that Arg may be an effective treatment for diabetes and obesity [12,13]. Results from cellular and animal experiments have shown that Arg can be metabolized to produce ornithine and promote wound healing, which has also been observed in burn patients [14,15]. Moreover, Arg promotes intestinal recovery. It was demonstrated that Arg activates mTOR, MAP kinase, and ribosomal signaling in enterocytes, stimulating protein synthesis and improving the intestinal mucosa [16,17]. Zhang et al. found that arginine intervention significantly ameliorated the *Clostridium-perfringens*-induced inflammatory response via the JAK-STAT pathway in in vivo and in vitro experiments [18]. Furthermore, Shah et al. found that arginine intervention significantly reduced the risk of necrotizing enterocolitis in neonates compared to the placebo group [19].

Our previous work demonstrated that barley leaf (BL) ameliorates *Citrobacter-rodentium* (CR)-induced colitis [20]. To further reveal the mechanism by which BL ameliorates CR-induced colitis, in this study, we explored the effect of BL on the metabolic profile of mice using non-targeted metabolomics techniques. Our results indicated that BL intervention significantly enriches arginine production and that arginine intervention significantly ameliorates CR-induced gut microbiota disorders and intestinal inflammation in a dose-dependent manner.

## 2. Materials and Methods

### 2.1. Animals

The mice utilized in this study were 4–6-week-old male C3H/HeN mice purchased from Beijing Vital River Laboratory Animal Technology Co., Ltd., (Beijing, China). Following one week of acclimatization, the mice were randomly divided into four groups: CD, CD+CR, AL+CR, and AH+CR (*n* = 10 per group). The AL+CR group was gavaged with low-dose arginine solution (1 g/kg·bw); the AH+CR group was gavaged with high-dose arginine solution (2 g/kg·bw), while the CD and CD+CR groups were gavaged with equal volumes of PBS. After gavage for 3 weeks, the mice in the CD+CR, AL+CR, and AH+CR groups were infected with CR to prepare a colitis model. The macronutrient composition of the diet is shown in Supplementary Table S1. (Ethics reference number: AW32602202-4-1).

### 2.2. CR Infection

Mice were infected with 1 × 10^9^ colony-forming units (CFUs)/mouse of the CR strain DBS 100 (ATCC 51459) to cause bacterial colitis. Briefly, the sterile Luria–Bertani medium was injected into a single CR colony grown on a fresh MacConkey agar plate (Solarbio, Beijing, China) and shaken overnight at 37 °C [21]. Mice were infected after three weeks of being fed a chow diet.

### 2.3. Determination of CR Load

Fresh stool pellets were collected, weighed, and homogenized using a BeadMill 24 benchtop bead-based homogenizer (Servicebio, Beijing, China) on days 1, 4, 7, and 10 following infection. The sample homogenates were then serially ten-fold diluted on MacConkey agar, and CR colonies were counted the next day. The CR colony can be distinguished by its distinctive features, which revolve around a red center and a white edge. 

### 2.4. Disease Activity Index (DAI)

The severity of colitis was evaluated by using the DAI score. Briefly, DAI was calculated by weight loss, stool consistency, and general health status, as previously described [22]. The DAI scoring scale is shown in Supplementary Table S2.

### 2.5. Histological Staining

After being fixed in the 4% formalin solution, the colonic tissues were embedded in paraffin. Samples were then cut into sections and stained with hematoxylin and eosin (H&E). As previously mentioned, tissue sections were estimated [22]. The histopathology scoring criteria are shown in Supplementary Table S3.

Samples were stained with the Alcian blue–periodic acid Schiff (AB-PAS) Stain Kit (Solarbio, Beijing, China) for goblet cell analysis. Image J software was used to perform image analysis.

### 2.6. RT-qPCR

Briefly, total RNA was extracted from the colon samples utilizing the Trizol reagent (Invitrogen, Waltham, MA, USA) and reversed into cDNA according to the instructions of the FastQuant RT Kit (TianGen, Beijing, China). RT-qPCR was performed using SYBR Real-time PCR Kit (Takara, Japan) by a LightCycler 480 Real-Time PCR system (Roche, Basel, Switzerland). Data were analyzed by the 2^−ΔΔCT^ method. The sequences of primers used in RT-qPCR are listed in Table S4.

### 2.7. 16S rRNA Gene Sequencing

The microbial diversity sequencing approach is as described in previous articles [20]. All raw sequence data were deposited in the NCBI Short Read Archive database under the Bioproject accession number PRJNA944610.

### 2.8. Non-Targeted Metabolomics

Mouse colon tissues were placed in centrifuge tubes, and metabolites were extracted by adding grinding beads and an extract containing an internal standard (L-2-chlorophenylalanine); quality control (QC) samples were prepared to investigate the reproducibility of the whole analysis process, and then the samples were detected by the ultra-performance liquid chromatography–tandem Fourier transform mass spectrometry UHPLC-Q Exactive system. After loading, the raw data were imported into Progenesis QI (Waters Corporation, Milford, MO, USA) software for processing, and the mass spectrometry information was also shared with the metabolic public databases HMDB (http://www.hmdb.ca/, accessed on 23 July 2021) and Metlin (http://metlin.scripps.edu/, accessed on 23 July 2021), and the Majorbio’s own library was matched to obtain metabolite information, after which the matrix data were preprocessed to obtain the data matrix for subsequent analysis. Principal component and orthogonal partial least squares discriminant analyses were performed with the R package ropls; in addition, Student’s *t*-test and difference multiplier analyses were performed.

### 2.9. Arginine and Polyamine Determination

Take an appropriate amount of sample in a centrifuge tube, and add 80% methanolic water containing 1% formic acid, vortex, and sonicate. Centrifuge (4 °C, 15,000 r/min, 5 min) and take the supernatant over the filter membrane containing H5 purification powder on the machine for detection. The data acquisition system included high-performance liquid chromatography (HPLC, SHIMADZU-20A) and tandem mass spectrometry (MS/MS, applied biosystems 6500 quadrupole trap). The mass spectrometry data were processed using the software Analyst, and the content was calculated using the standard curve.

### 2.10. Statistics

Data are presented as MEAN ± SEM. A two-tailed unpaired Student’s *t*-test was used to assess whether there were significant differences between the two groups. In more than two groups, statistical significance was determined using one-way or two-way analysis of variance (ANOVA), followed by the Duncan test, and the Bonferroni statistical test was used post hoc. A *p*-value < 0.05 was considered statistically significant.

## 3. Results

### 3.1. Effect of BL on Colonic Tissue Metabolites

**Figure 1:**
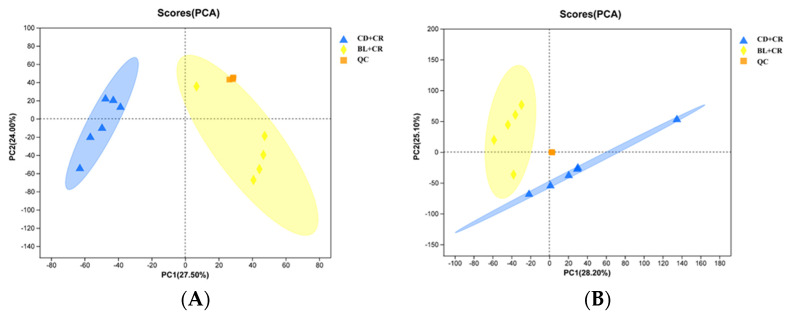
PCA analysis of metabolites in mouse colon tissue in different modes ((**A**): negative ion mode; (**B**): positive ion mode). CD+CR, model group; BL+CR, barley leaf intervention group; QC, quality control. Blue, CD+CR group; Yellow, BL+CR group; Orange, QC.

To investigate the effect of dietary supplementation with BL on colonic tissue metabolites in mice, we analyzed the metabolite composition differences in mouse colonic tissues based on a non-targeted metabolomics technique with LC-MS/MS platform. For statistical analysis, we used a combination of positive and negative ion patterns to ensure the integrity and credibility of the metabolic data. As shown by the principal component analysis, the metabolites had good clustering among the groups in the negative ion mode, and the clusters were significantly separated between the CD+CR and BL+CR groups; in the positive ion mode, the principal component analysis also showed that the two groups of samples formed independent regions that did not overlap each other (Figure 1). In conclusion, it is clear that dietary supplementation with BL had a significant effect on the metabolism of mice.

**Figure 2:**
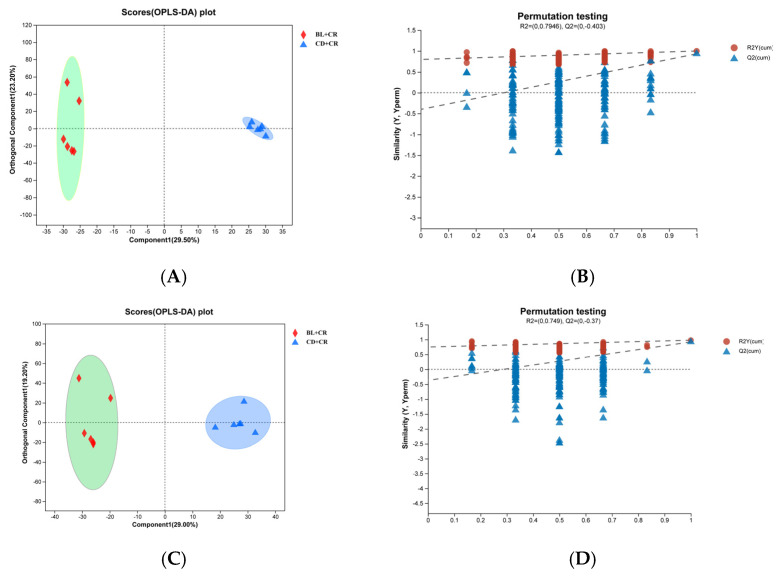
OPLS-DA analysis of mouse colon tissue metabolites in different modes. (OPLS-DA score plot (**A**) and permutation test (**B**) in negative ion mode; OPLS-DA score plot (**C**) and permutation test (**D**) in positive ion mode). CD+CR, model group; BL+CR, barley leaf intervention group. Red, BL+CR group; Blue, CD+CR group.

To further analyze the differences between the samples, we performed orthogonal partial least squares discriminant analysis (OPLS-DA) on the obtained data matrix. As shown in Figure 2, the two groups in the OPLS-DA score plot form independent regions with significant differences in the negative or positive ion model; the R^2^Y and Q^2^ values are close to 1, indicating that the model is stable and reliable. Furthermore, the regression lines of R^2^Y and Q^2^ in the OPLS-DA permutation test increase with the permutation retention, demonstrating that the model does not appear to be overfitted. We also performed a partial least squares discriminant analysis (PLS-DA). Consistent with the OPLS-DA, the PLS-DA results also showed that the two groups of mouse metabolite clusters showed a significant separation (Supplementary Figure S1).

### 3.2. KEGG Functional Enrichment Analysis of Differential Metabolites for BL Intervention

**Figure 3:**
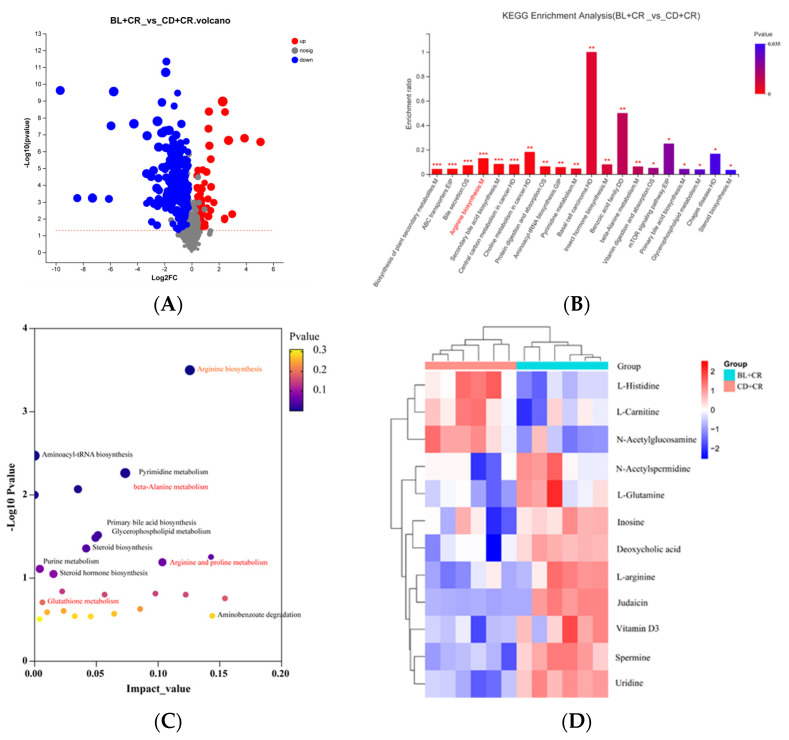
Differential metabolite analysis ((**A**): Volcano map; (**B**): differential metabolite KEGG functional enrichment analysis; (**C**): differential metabolite KEGG topology analysis; (**D**): heatmap analysis). CD+CR, model group; BL+CR, barley leaf intervention group. * *p* < 0.05, ** *p* < 0.01, *** *p* < 0.001.

Based on the variable importance (VIP) and Student’s *t*-test *p*-value obtained from the OPLS-DA model, we selected metabolites with VIP > 1 and *p* < 0.05 as differential metabolites. After the screening, there were 175 differential metabolites between the two groups (Figure 3). Afterward, KEGG functional enrichment analysis and KEGG topology analysis were performed on the differential metabolites. The results showed that these differential metabolites were mainly involved in arginine metabolism, glutamine metabolism, secondary bile acid metabolism, β-alanine metabolism, pyrimidine metabolism, glycerophospholipid metabolism, and steroid biosynthesis, among which arginine metabolism was the most significant in KEGG pathway annotation (Figure 3B,C). Then, we performed the heatmap analysis for both groups of differential metabolites. As shown in Figure 3D, dietary supplementation with BL significantly increased the levels of L-arginine, vitamin D3, inosine, L-glutamine, spermine, and uridine in mouse colonic tissues compared with the CD+CR group.

### 3.3. BL Intervention Enriches Arginine Production in Mice

**Figure 4:**
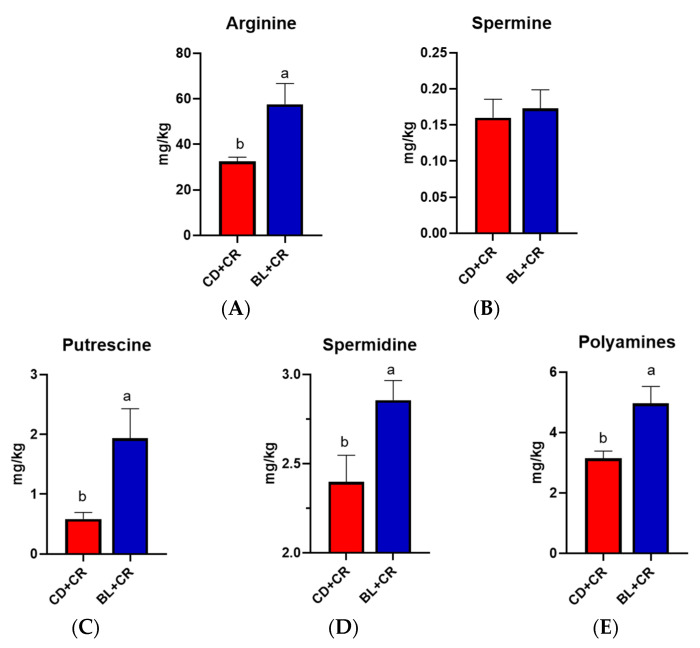
Determination of arginine and polyamines in mouse colonic tissue ((**A**): L-arginine; (**B**): spermine; (**C**): putrescine; (**D**): spermidine; (**E**): polyamines). *n* = 7, different letters indicate significant differences (*p* < 0.05). CD+CR, model group; BL+CR, barley leaf intervention group.

**Figure 5:**
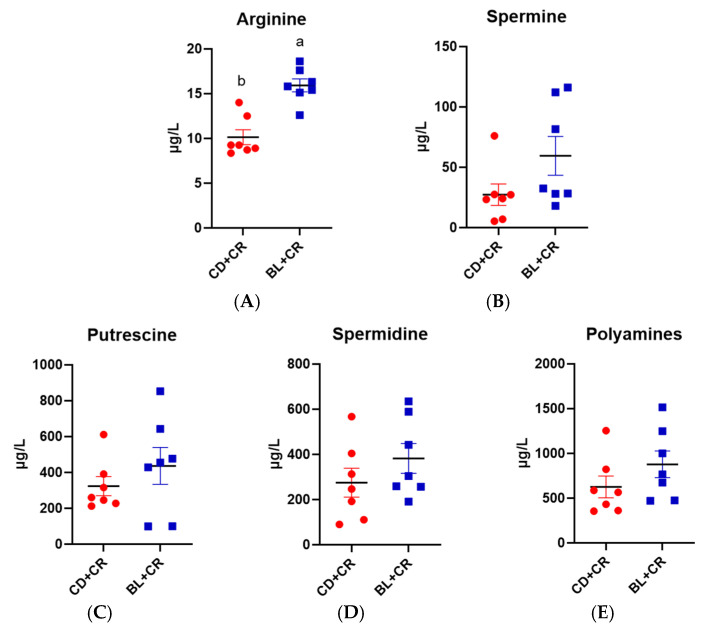
Determination of arginine and polyamines in mouse serum. ((**A**): L-arginine; (**B**): spermine; (**C**): putrescine; (**D**): spermidine; (**E**): polyamines). *n* = 7, different letters indicate significant differences (*p* < 0.05). CD+CR, model group; BL+CR, barley leaf intervention group.

From the non-targeted metabolomics results, it is clear that dietary supplementation of BL increased the content of arginine in mice. To further determine the effect of BL on mouse metabolism, we examined the content of arginine and polyamines in mouse colonic tissues and serum using the LC-MS/MS method. As shown in Figure 4, dietary supplementation with BL significantly increased the content of arginine in mouse colonic tissues compared with mice in the CD+CR group, and also significantly increased the content of spermidine and putrescine in the mouse colon, but had no effect on the change in spermine content. In addition, we assayed the content of arginine and polyamines in the serum of mice, and the results are shown in Figure 5. From the serum assay results, it can be seen that dietary supplementation of BL significantly increased the serum content of arginine in mice, but there was no significant difference in the content of spermine, spermidine, and putrescine. In conclusion, dietary supplementation of BL can increase the content of arginine in mice.

### 3.4. Arginine Improves CR-Induced Colitis

**Figure 6:**
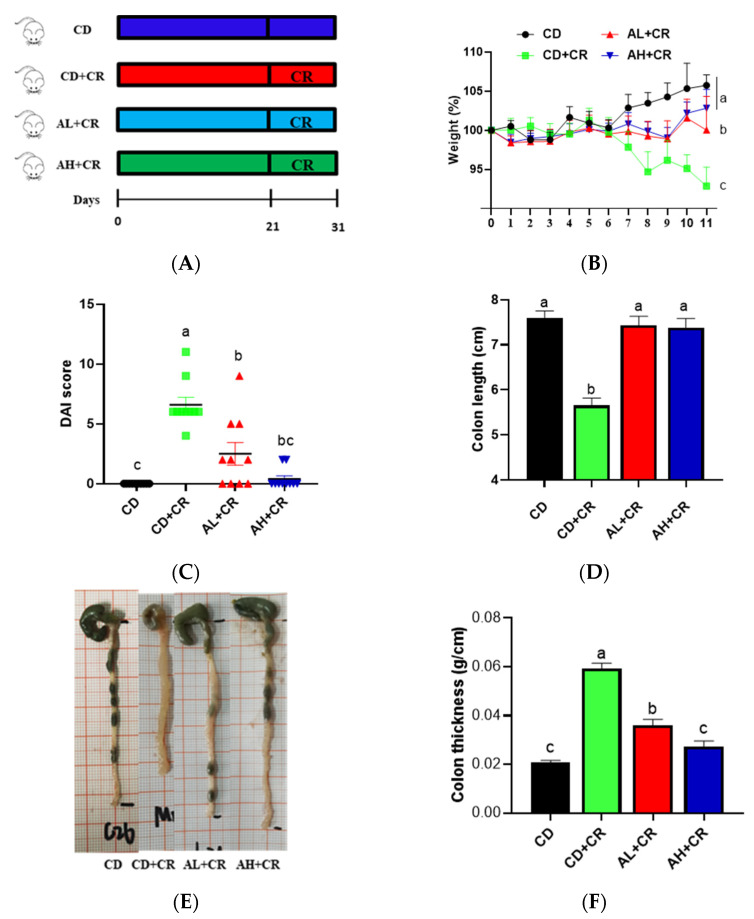
Arginine ameliorates CR-induced colitis ((**A**): experimental design; (**B**): weight loss; (**C**): disease activity index; (**D**): colon length; (**E**): representative map of overall appearance of intestinal tissues; (**F**): colon thickness). *n* = 10, different letters indicate significant differences (*p* < 0.05). CD, control group; CD+CR, model group; AL+CR, 1 g/(kg·bw) arginine intervention; AH+CR, 2 g/(kg·bw) arginine intervention.

From the previous section, it is known that BL promotes arginine content, so can arginine improve CR-induced colitis? To test this hypothesis, we designed the following animal experiments (Figure 6A). Compared with the CD group, CR resulted in colitis symptoms such as weight loss, elevated DAI index, shortened colon, wrinkled cecum, and swollen colonic wall in mice; in comparison with the CD+CR group, mice in the AL+CR and AH+CR groups showed a significant improvement in weight loss, elevated DAI index, and shortened colon (Figure 6B–F). Interestingly, mice in the AH+CR group also showed significant differences in body weight loss and colon thickness compared with the AL+CR group(Figure 6B,F), suggesting that the ameliorative effect of arginine might be dose-dependent.

### 3.5. Arginine Ameliorates Histopathological Damage Caused by CR

**Figure 7:**
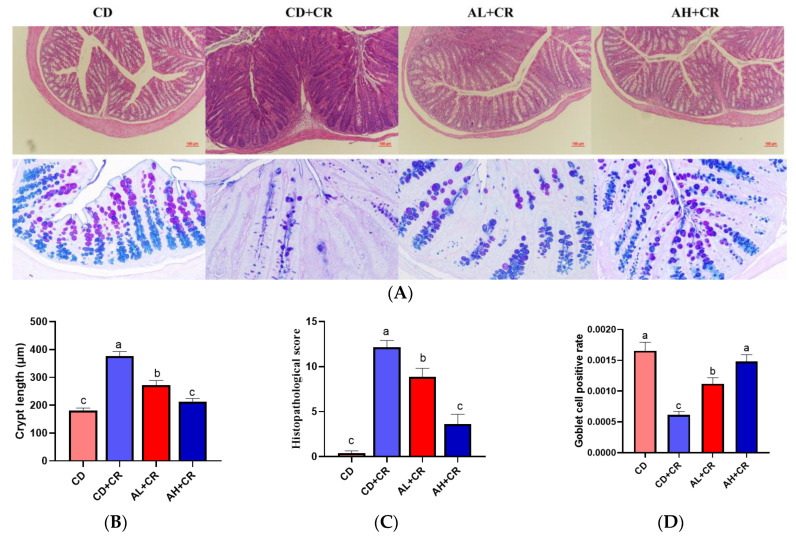
Arginine ameliorates intestinal tissue damage due to CR ((**A**): representative colon tissue H&E section staining and Alcian blue–periodic Schiff stained section; (**B**): crypt length; (**C**): pathology score; (**D**): goblet cells statistical analysis). *n* = 8, different letters indicate significant differences (*p* < 0.05). CD, control group; CD+CR, model group; AL+CR, 1 g/(kg·bw) arginine intervention; AH+CR, 2 g/(kg·bw) arginine intervention.

To further investigate the ameliorative effect of arginine on CR-induced colitis, we observed and analyzed the mouse colonic tissue microstructure. As shown in Figure 7A, the H&E results showed that CR caused the intestinal barrier integrity loss, and a large number of inflammatory cells infiltrated intestinal tissues and induced intestinal crypt abnormal proliferation in mice compared with the CD group; in comparison with the CD+CR group, the intestinal tissue damage was significantly improved in the AL+CR and AH+CR groups. Notably, the AL+CR and AH+CR groups also showed significant differences in intestinal histopathology, which is consistent with the above-described results (Figure 6). In addition, the AB-PAS results showed that CR resulted in a dramatic reduction in goblet cells in mice compared to the CD group. In contrast, arginine intervention significantly ameliorated the CR-induced goblet cell deficiency and was consistent with the H&E staining results. Notably, the positive rate of goblet cells in the AH+CR group was significantly higher than that in the AL+CR group (Figure 7A,B). In conclusion, arginine improved intestinal tissue damage caused by CR, and the improvement effect on pathological damage was also dose-dependent.

### 3.6. Arginine Inhibits the Proliferation of CR and the Expression of Virulence Factors

**Figure 8:**
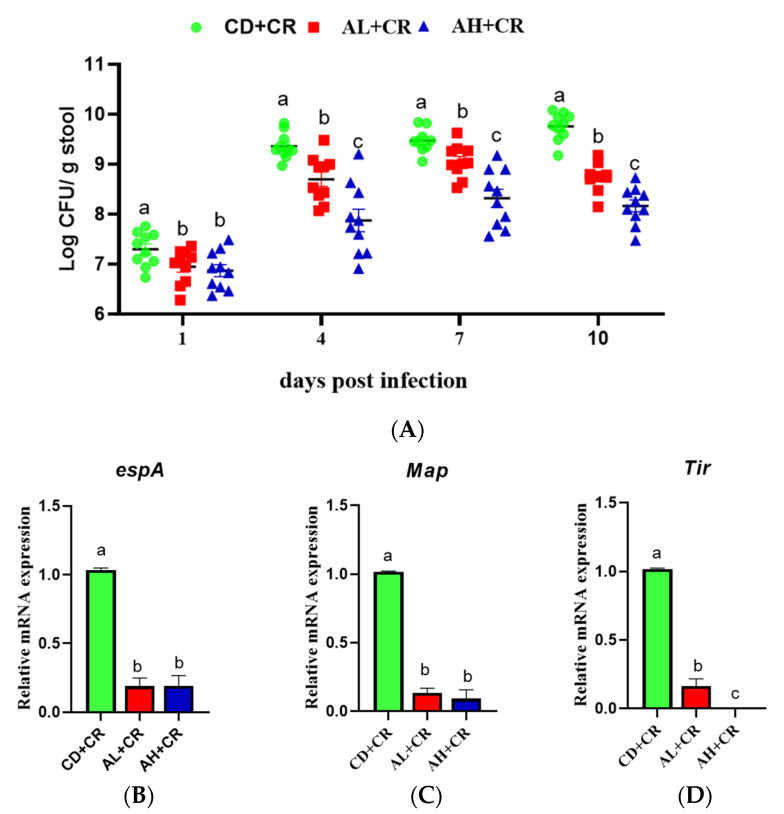
Arginine inhibits the proliferation of CR and the expression of virulence factors ((**A**): the CR burden in the feces of mice on days 1, 4, 7, 10 after infection, *n* = 10; (**B**): the relative expression of espA, *n* = 8; (**C**): the relative expression of Map, *n* = 8; (**D**): the relative expression of Tir, *n* = 8). Different letters indicate significant differences (*p* < 0.05). CD, control group; CD+CR, model group; AL+CR, 1 g/(kg·bw) arginine intervention; AH+CR, 2 g/(kg·bw) arginine intervention.

CR’s massive proliferation in the intestinal lumen depends on its virulence factor expression, so we explored the effect of arginine on CR proliferation and its virulence factor expression. As shown in Figure 8A, CR proliferated rapidly in the intestinal lumen after infection; the CR load in mice feces was significantly lower in the AL+CR and AH+CR groups compared with the CD+CR group. Interestingly, the CR load in mice feces was significantly lower in the AH+CR group than in the AL+CR group on days 4, 7 and 10 after infection, indicating that arginine significantly inhibited the proliferation of CR, and this inhibitory effect was dose-dependent. Next, we examined the expression of CR virulence factors *espA*, *Map*, and *Tir* using the RT-qPCR technique. The results showed that CR’s virulence factor expression was significantly lower in mice in the AL+CR and AH+CR groups compared to the CD+CR group, and notably, no expression of the *Tir* gene, which is essential for CR’s formation of A/E lesions and consequently TMCH [23,24], was detected in mice under high-dose arginine intervention, which may partially explain the significant superiority of high-dose arginine over low-dose arginine in improving colitis (Figure 8D).

### 3.7. Arginine Ameliorates CR-Induced Gut Microbiota Disorders

**Figure 9:**
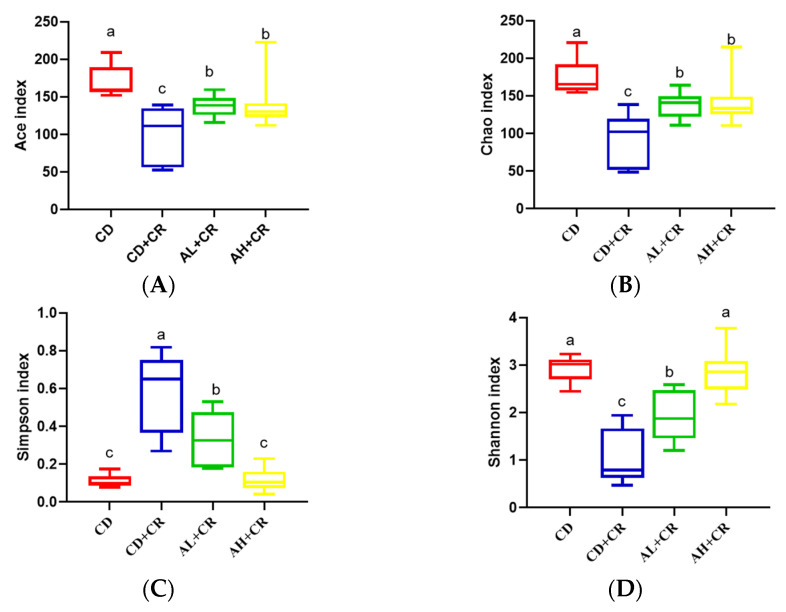
Arginine ameliorates gut microbiota disorders caused by CR ((**A**): Ace index; (**B**): Chao index; (**C**): Simpson index; (**D**): Shannon index). CD, control group; CD+CR, model group; AL+CR, 1 g/(kg·bw) arginine intervention; AH+CR, 2 g/(kg·bw) arginine intervention. Different letters indicate significant differences (*p* < 0.05).

Consistent with previously reported findings [20,25], CR infestation led to a decrease in mouse intestinal flora diversity and richness (the Ace and Chao indices characterize community richness; the Shannon and Simpson indexes characterize community diversity), whereas compared with the CD+CR group, arginine treatment significantly increased the Ace and Chao index while significantly inhibiting Simpson index, which was consistent with the results of the Shannon index. Compared with the AL+CR group, there was no significant difference in the Ace index and Chao index in the AH+CR group, but there were significant differences in the Simpson index and Shannon index, indicating that different doses of arginine affected the intestinal flora diversity in mice (Figure 9). In conclusion, arginine significantly improved the reduction in intestinal microbial richness and diversity caused by CR.

**Figure 10:**
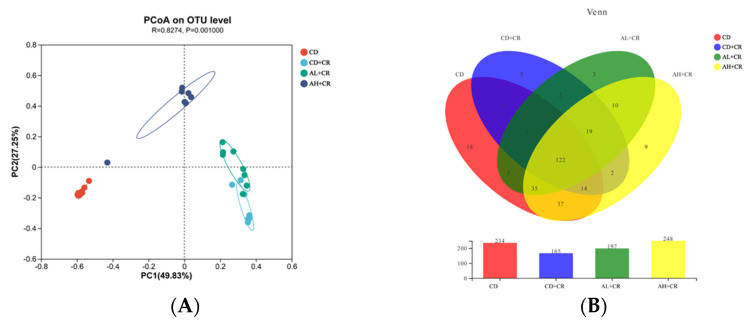
Beta diversity analysis ((**A**): PCoA analysis; (**B**): Venn analysis). CD, control group; CD+CR, model group; AL+CR, 1 g/(kg·bw) arginine intervention; AH+CR, 2 g/(kg·bw) arginine intervention.

To further investigate the effect of arginine on mouse intestinal flora, we examined the beta diversity. PCoA analysis showed that each mouse intestinal flora group formed its own clustering region. There was only minimal overlap between the clusters of the AL+CR and CD+CR groups, while the clusters of the AH+CR and CD+CR groups were significantly separated, indicating that different doses of arginine intervention on mouse flora were different, which might be related to its different degrees of improvement of colitis (Figure 6 and Figure 7). Venn analysis showed that there were 122 OTUs shared among the four groups. Compared with the CD group, there were 2 OTUs in the CD+CR group, while there were 37 OTUs in the AH+CR group and 5 OTUs in the AL+CR group. The number of OTUs shared between the AL+CR and CD+CR groups was 2, and the number of OTUs shared between the AH+CR and CD+CR groups was 2 (Figure 10B). In conclusion, arginine significantly improved the intestinal flora disorder caused by CR.

### 3.8. Effect of Arginine on the Gut Microbiota’s Composition and Structure

To further investigate the effect of arginine on the gut microbiota composition, we analyzed the mouse intestinal microorganisms at the phylum level and the genus level. At the phylum level, CR infection resulted in a significant increase in the relative abundance of the pathogenic phylum Proteobacteria compared to the CD group; while arginine intervention significantly suppressed the Proteobacteria relative abundance (Supplementary Figure S2). 

**Figure 11:**
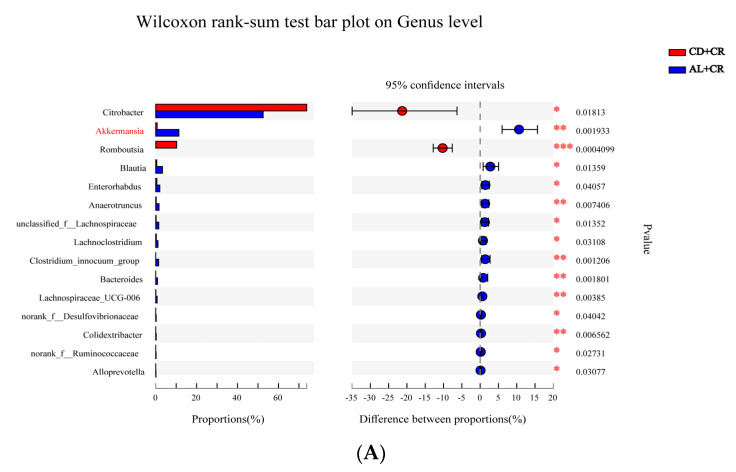

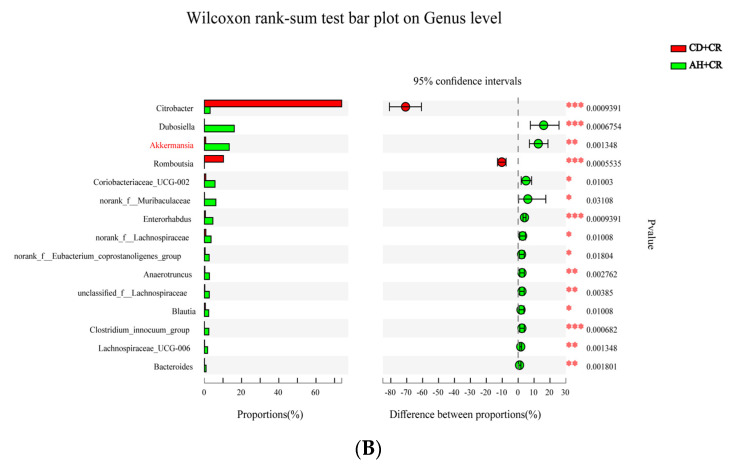
Effect of arginine intervention on the genus level of gut microbiota. ((**A**): Analysis of differences in genus levels between CD+CR and AL+CR groups; (**B**): analysis of differences in genus levels between CD+CR and AH+CR groups). CD+CR, model group; AL+CR, 1 g/(kg·bw) arginine intervention; AH+CR, 2 g/(kg·bw) arginine intervention. Red, CD+CR group; Blue, AL+CR group; Green, AH+CR group. * *p* < 0.05, ** *p* < 0.01, *** *p* < 0.001.

As shown in Figure S3, there were significant variations in the intestinal flora of each group of mice at the genus level in terms of composition structure. To further analyze the genera in which arginine intervention caused significant differences in the intestinal flora of mice, we analyzed the gut microbiota using the Kruskal–Wallis H test. As shown in Figure 11, the relative content of CR in the AL+CR and AH+CR groups was significantly lower compared to the CD+CR group, and the abundance of CR in the AH+CR group was inferior compared to the AL+CR group, which was consistent with our previous results of the fecal dilution plate (Figure 8A), again indicating that arginine significantly inhibited the proliferation of CR in the intestinal lumen, and this inhibition showed a dose-dependent effect; in addition to inhibiting CR, arginine intervention significantly inhibited the relative abundance of *Romboutsia*. Furthermore, when compared with the CD+CR group, the relative abundance of *Akkermansia*, *Blautia*, *Enterorhabdus*, *Anaerotruncus*, *Bacteroides*, *unclassified_f_Lachnospiraceae*, *Lachnospiraceae_UCG-006*, *Clostridium_innocuum_group*, norank_f_Desulfovibrionaceae, Lachnoclostridium, and *norank_f_Ruminococcaceae* in the AL+CR group were significantly higher, and the relative contents of *Dubosiella*, *Akkermansia*, *Coriobacteriaceae_UCG-002*, *norank_f_Muribaculaceae*, *Enterorhabdus*, *norank_f_Lachnospiraceae*, *Anaerotruncus*, *unclassified_f_Lachnospiraceae*, *Blautia*, and *Lachnospiraceae_UCG-006* in the AH+CR group were significantly higher compared with the CD+CR group. It can be seen that different doses of arginine intervention can jointly increase the abundance of *Akkermansia*, *Enterorhabdus*, *unclassified_f_Lachnospiraceae*, *Blautia*, and *Lachnospiraceae_UCG-006*, among which *Akkermansia* was the most enriched genus in both interventions. 

### 3.9. Gut Microbiota Function Prediction

PICRUSt normalized the mouse intestinal flora OTUs, and then obtained the corresponding KEGG Ortholog (KO)-related information based on the greengene id, which in turn can be compared with the KEGG database to obtain the related information of the pathways as well as to calculate the abundance of each functional class based on the OTU abundance. As shown in Figure S4, among the predicted relevant pathways, there are several pathways associated with resistance to pathogenic bacteria, including biofilm formation–Escherichia coli, bacterial invasion of epithelial cells, pathways in cancer, pathogenic Escherichia coli infection, shigellosis, Yersinia infection, etc. In addition, there are several metabolism-related pathways, such as biosynthesis of amino acids, pyruvate metabolism, nitrogen metabolism, arginine biosynthesis, secondary bile acid biosynthesis, and lysine biosynthesis, suggesting that dietary arginine supplementation might improve the gut microbiota’s composition and metabolism to resist colitis caused by pathogenic bacteria.

## 4. Discussion

IBD has now become a global public health challenge; however, the conventional pharmacological treatments are less effective and have greater side effects [3]. Thus, there is an urgent need to develop new treatment modalities for IBD. Our previous results showed that BL significantly improved CR-induced colitis, but its mechanism remains elusive [20]. To further reveal its action mechanism and also to provide a theoretical basis for food intervention in the disease, in this study, we used non-targeted metabolomics techniques to search for potentially effective metabolites produced by BL intervention and conducted experimental studies on the effects of differential metabolites.

To ensure the integrity and credibility of the metabolic data, we used a combination of positive and negative patterns for statistical analysis. PCA and OPLS-DA score plots revealed that BL intervention had a significant effect on mouse metabolic profile (Figure 1 and Figure 2), and we screened a total of 175 differential metabolites based on VIP > 1 and *p* < 0.05. The KEGG pathway enrichment analysis of the differential metabolites revealed that the metabolism-related pathways affected by barley leaf intervention included the Biosynthesis of plant secondary metabolites, arginine biosynthesis, secondary bile acid biosynthesis, pyrimidine metabolism, insect hormone biosynthesis, beta-alanine metabolism, primary bile acid biosynthesis, glycerophospholipid metabolism, and steroid biosynthesis, among which the arginine biosynthesis was the second most significant only to biosynthesis of plant secondary metabolites (Figure 3B). Furthermore, the KEGG topological results indicated that the impact value and Log10 *p*-value of arginine biosynthesis were the highest (Figure 3C). The larger the impact value, the more important the pathway is. Combining Figure 3B,C, we consider that arginine biosynthesis is the most significant metabolic pathway for barley leaf to improve *Citrobacter-rodentium*-induced colitis, and in the heatmap analysis, we also found that arginine is significantly elevated (Figure 3D), while arginine is the predominant substrate for polyamine metabolism. Thus, we think that arginine is the most important metabolite for barley leaf to exert an improving effect on colitis. Therefore, we examined the arginine content in mice. In comparison with the CD+CR group, dietary supplementation with BL significantly increased the content of arginine in the mouse colon, which was consistent with the detection of serum, indicating that BL intervention could significantly enrich arginine (Figure 3D, Figure 4A and Figure 5A). Furthermore, arginine is the predominant substrate for polyamine metabolism, and Weiss et al. found that polyamine metabolism rates were significantly lower in IBD patients compared to healthy individuals [26]. Thus, we also examined the levels of polyamines in mouse serum and colonic tissues. Our results showed that BL intervention significantly increased spermidine and putrescine levels in colonic tissues, while it did not have a significant effect on serum polyamine levels (Figure 4B–E and Figure 5B–E), probably due to the fact that polyamines are mainly found in areas of rapid tissue renewal, such as intestinal epithelial cells [27]. In addition, polyamines have been well-documented as modulating host immune responses, influencing cell growth and development, and accelerating tissue damage repair [28,29], which might partially explain why high arginine doses improve CR-induced colitis significantly better than low arginine doses (Figure 6 and Figure 7).

Arginine is a semi-essential amino acid that is depleted under physiologically stimulating conditions [30]. In clinical practice, arginine is often used to treat blood ammonia toxicity and male infertility due to insufficient semen production and sperm deficiency [31]. It has been reported that arginine levels are significantly lower in IBD patients compared to healthy individuals [32,33]. Thus, could BL intervention of arginine enrichment ameliorate CR-induced colitis? As a result, the effect of different arginine doses on CR-induced colitis was investigated in this study. It has been reported that oral administration of 20 g of arginine per day in adults had no adverse effects [34], whereas the equivalent dose of 2 g/(kg·bw) in this study was 15.4 g/(70 kg·d) based on the “human-animal body surface area conversion”, which was much lower than 20 g/(70 kg·d). Consistent with previous reports, CR infection resulted in significant colitis manifestations, such as a reduced body weight, shortened colon, and wrinkled cecum [20,22], whereas arginine intervention significantly ameliorated CR-induced colitis, and this ameliorative effect was dose-dependent (Figure 6). Transmissible murine crypt hyperplasia (TMCH) is the hallmark feature of CR-infected mice, and is triggered by CR using its type III secretion system to inject virulence proteins into enterocytes [21]. As seen in the H&E results, arginine intervention significantly improved CR-caused TMCH, and the AB-PAS results confirmed the H&E findings that arginine improved goblet cell deficiency caused by CR (Figure 7). Intriguingly, the ameliorative effect of arginine on CR-induced TMCH also showed a dose-dependent effect. In conclusion, arginine dramatically modified CR-induced colitis in a dose-dependent manner. 

Pathogens rely on their virulence factors to acquire exclusive ecological niches where they proliferate and eventually lead to disease. It has been demonstrated that *Tir* is the key gene in CR causing A/E lesions, which in turn are required to cause TMCH [35]. Analysis of CR virulence factor expression showed that high-dose arginine had a remarkably greater inhibitory effect on *Tir* expression than low-dose arginine (Figure 8D); in addition, dilution coating of stool samples and microbial diversity analysis demonstrated that different concentrations of arginine inhibited CR proliferation at different levels (Figure 8A and Figure 11), which partly explains the dose-dependent effect of arginine on the amelioration of CR-caused colitis. 

It has been demonstrated that the intestinal flora resisted the invasion of foreign bacteria and the expansion of pathogenic bacteria, a phenomenon known as “colonization resistance” [36]. From the PCoA plots, it was observed that the different doses of arginine intervention also had variable effects on CR-induced gut microbiota disorders in mice, which may be related to its different degrees of improvement of colitis. Numerous clinical and animal studies have shown that Proteobacteria could facilitate the development of IBD by triggering inflammation and altering the intestinal flora [37], and our experimental results showed arginine treatment could significantly decrease the relative abundance of Proteobacteria compared to the CD+CR group (Figure S2). At the genus level, arginine dramatically increased the relative abundance of *Akkermansia* as well as significantly inhibiting CR (Figure 11). *Akkermansia* is widely distributed in the intestinal mucus layer and could utilize mucin to produce SCFAs, accounting for about 3% of the intestinal flora, and it has been demonstrated to have probiotic potential. Our previous research indicated that both live and inactivated *Akkermansia* dramatically improved DSS-induced colitis [38]. Furthermore, *Akkermansia* has been shown to significantly increase tight junction protein expression, promote mucin secretion such as Muc2, and improve the intestinal barrier environment [39]. Meng et al. revealed that *Akkermansia muciniphila* could inhibit the viability of human colorectal cancer LS174T cells through the TRAIL-mediated apoptosis pathway [40]. Bian et al. discovered that pro-inflammatory cytokines and other injurious factors were negatively correlated with *Akkermansia*, *Ruminococcaceae*, and *Rikenellaceae* in the DSS-induced ulcerative colitis mouse model, confirming that *A. muciniphila* treatment could improve mucosal inflammation through microbial–host interactions or by improving the microbial community to ameliorate mucosal inflammation [41].

Apart from *Akkermansia*, arginine also dramatically increased the relative abundance of *Blautia*, *Enterorhabdus*, and *Lachnospiraceae*. There is a positive correlation between *Blautia* abundance and beneficial butyrate production, and in IBD patients, *Blautia* abundance is reduced [42], and Bajaj et al. found that *Blautia* could mediate beneficial anti-inflammatory effects [43]. *Lachnospiraceae* is important in maintaining intestinal health. Multiple studies suggested that *Lachnospiraceae* might have an impact on metabolic syndrome, obesity, diabetes, liver diseases, and IBD. For example, Tannock et al. showed that the *Lachnospiraceae* level was significantly lower in IBD patients compared to healthy individuals [44]; Ma et al. found that glucose metabolism disorders in osteoporotic rats were caused by a reduction in *Lachnospiraceae* [45]; and Phannasorn et al. revealed that RBBO was able to inhibit biomarkers of liver and colon cancer in rats by forcing apoptosis, reducing inflammation, and increasing *Lachnospiraceae* [46]. Additionally, a recent study showed a significant negative association between *Enterorhabdus* and IBD [47]. Therefore, arginine could alleviate CR-induced colitis by modulating gut microbiota. Although we have explored BL interventions enriching arginine to ameliorate CR-induced colitis, fewer studies have addressed other metabolites in the KEGG metabolic pathway and fewer studies have investigated polyamine metabolites of arginine, which should be further explored in future studies.

## 5. Conclusions

In this study, our results demonstrated that dietary supplementation with barley leaf significantly enriched arginine and that arginine intervention significantly ameliorated CR-induced colitis symptoms such as a reduced body weight, shortened colon, wrinkled cecum, and swollen colon wall in mice; in addition, arginine intervention dramatically ameliorated CR-induced histopathological damage to the colon. The gut microbial diversity analysis showed that arginine intervention significantly decreased the relative abundance of CR and significantly increased the relative abundance of *Akkermansia*, *Blautia*, *Enterorhabdus*, and *Lachnospiraceae*, which modified the CR-induced intestinal flora disorder. Notably, arginine showed a dose-dependent effect on the improvement of colitis caused by CR. Our works provide a reference for the therapy and prevention of IBD.

## Supplementary Materials

The following supporting information can be downloaded at: https://www.mdpi.com/article/10.3390/nu15081890/s1, Figure S1: PLS-DA analysis of mouse colon tissue metabolites in different modes; Figure S2: Effect of arginine intervention on gut microbiota at the phylum level; Figure S3: Effect of arginine intervention on the genus level of gut microbiota; Figure S4: Pathways predicted by gut microbiota function to KEGG Level 3 levels; Table S1: The macronutrient composition of chow diet; Table S2: Disease activity index; Table S3: Histopathological scores; Table S4: Primer sequence.
