# Supplementary material for: Barley Leaf Ameliorates Citrobacter-rodentium-Induced Colitis through Arginine Enrichment
Source: Nutrients. 2023 Apr 14;15(8):1890. doi: 10.3390/nu15081890 (PMC10145403; doi:10.3390/nu15081890)
Supplement: Supplementary file 1 [file nutrients-15-01890-s001.zip › nutrients-2334842-supplementary.pdf]

## Supplemental file

# Barley leaf ameliorates Citrobacter rodentium-induced colitis through arginine enrichment

Yu Feng, Daotong Li, Chen Ma, Xiaosong Hu, Fang Chen*

**This file includes:**

Figure S1. PLS-DA analysis of mouse colon tissue metabolites in different modes;

Figure S2. Effect of arginine intervention on gut microbiota at the phylum level;

Figure S3. Effect of arginine intervention on the genus level of gut microbiota;

Figure S4. Pathways predicted by gut microbiota function to KEGG Level 3 levels;

Table S1. The macronutrient composition of chow diet;

Table S2. Disease activity index;

Table S3. Histopathological scores.

Table S4. Primer sequences

---

**Correspondence:** College of Food Science and Nutritional Engineering, National Engineering Research Center for Fruit and Vegetable Processing, Key Laboratory of Fruit and Vegetables Processing Ministry of Agriculture, En-gineering Research Centre for Engineering Vegetables Processing, Ministry of Education, China Agricul-tural University, Beijing 100083, China;

**E-mail:** chenfangch@sina.com; **Tel/Fax:** +86-10-62737654 ext 18.

# Figure S1

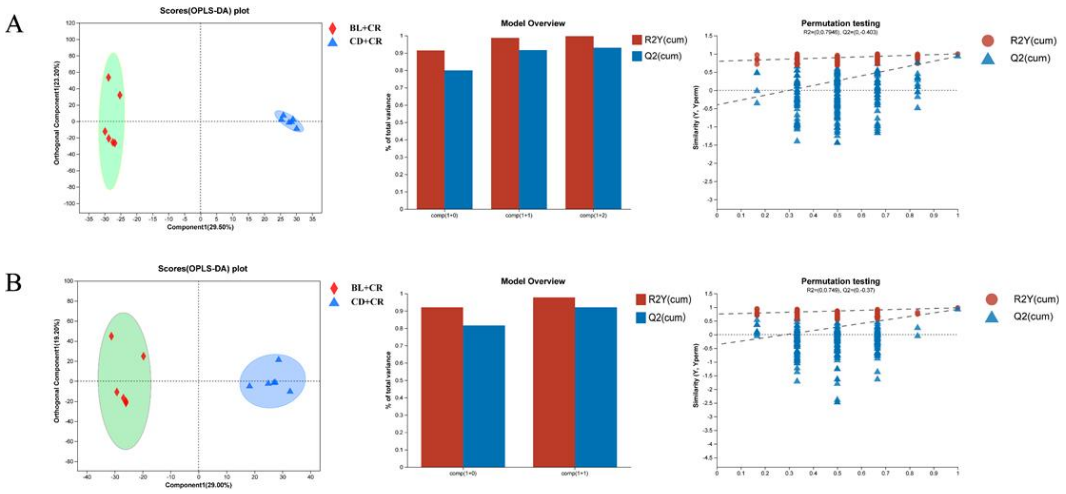


**Figure S1. PLS-DA analysis of mouse colon tissue metabolites in different modes.** (A: PLS-DA score plot and permutation test in negative ion mode; B: PLS-DA score plot and permutation test in positive ion mode)

# Figure S2

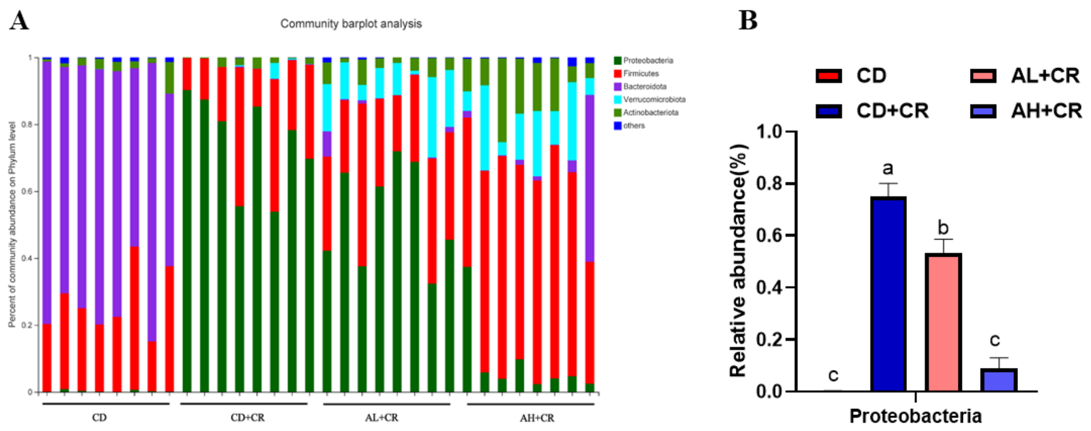


**Figure S2. Effect of arginine intervention on gut microbiota at the gate level** (A: Bar graph of community composition at the gate level; B: Relative content of Proteobacteria) All values are the mean ± SEM, n = 8 if not specified. a, b, c means in the same bar without a common letter differ at $P < 0.05$.

# Figure S3

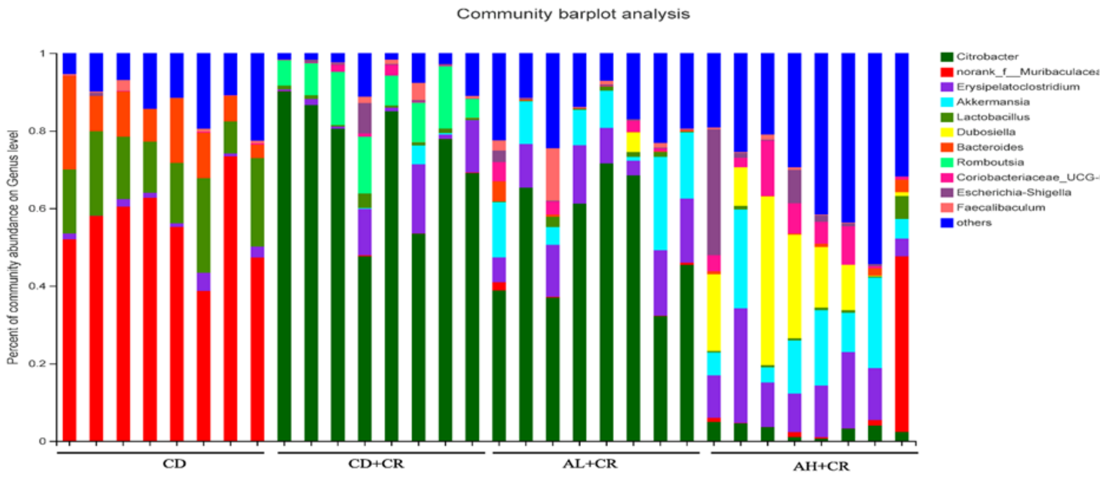


**Figure S3. Effect of arginine intervention on the genus level of gut microbiota.** Each bar represents the taxonomic composition and relative abundance of an individual mouse at the genus level.

# Figure S4

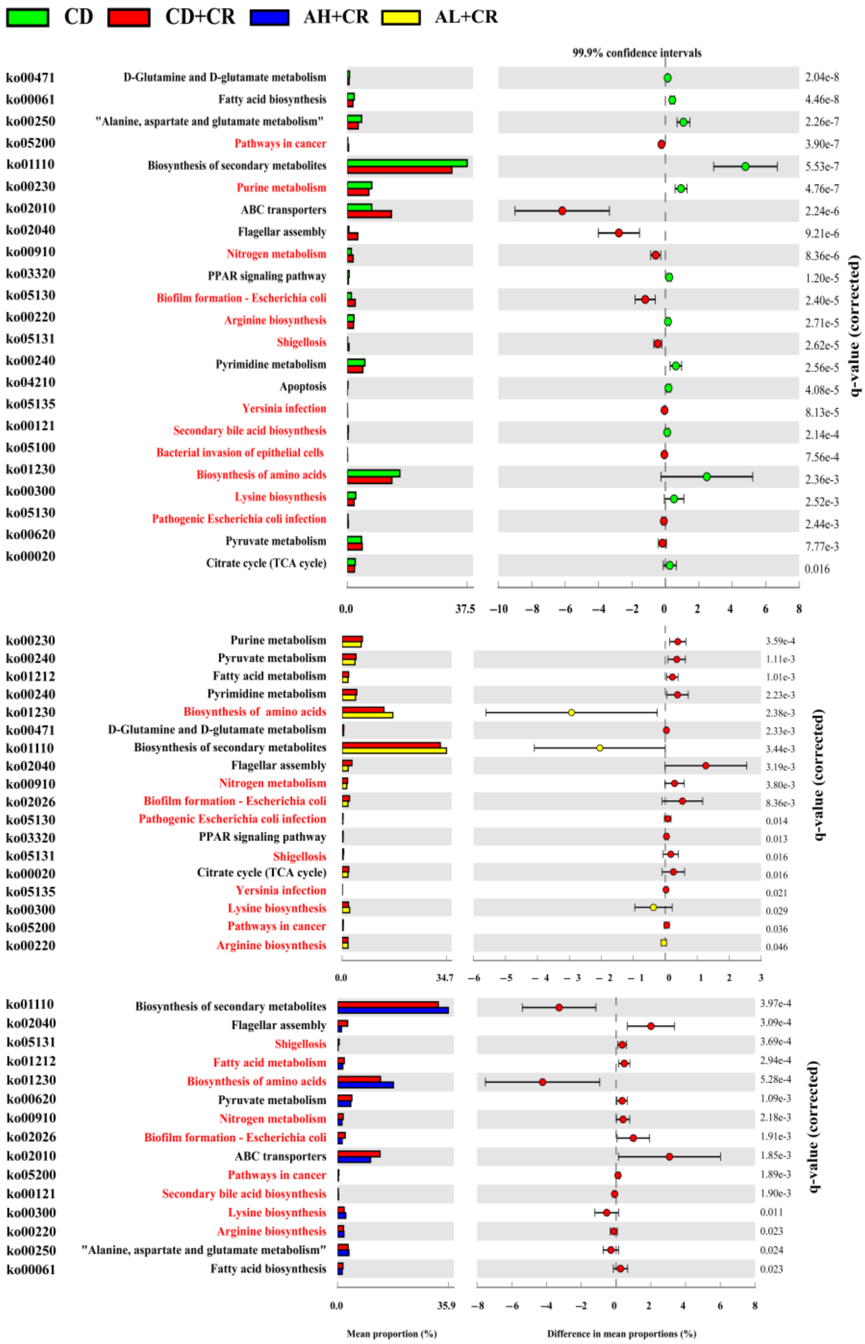


**Figure S4. Pathways predicted by gut microbiota function to KEGG Level 3 levels.**

# Table S1

**Table S1.** The macronutrient composition of chow diet

| Ingredients (g/kg of diet) | Chow Diet |
|---|---|
| Casein | 189.58 |
| L-Cysteine | 2.84 |
| Corn Starch | 298.59 |
| Maltodextrin | 33.18 |
| Sucrose | 331.77 |
| Cellulose | 47.40 |
| Soybean oil | 23.70 |
| Lard | 18.96 |
| Mineral Mix M1002 | 9.48 |
| DiCalcium Phosphate | 12.32 |
| Calcium Carbonate | 5.21 |
| Potassium Citrate | 15.64 |
| Vitamin mix V10001 | 9.48 |
| Choline Bitartrate | 1.90 |
| Total | 1000 |

# Table S2

**Table S2.** Disease activity index scores

| Score | Weight loss （%） | stool consistency | general health |
|---|---|---|---|
| 0 | 0 | Normal | Normal |
| 1 | 1-5 | | Mild |
| 2 | 6-10 | Loose | Moderate |
| 3 | 11-15 | | Severe |
| 4 | >16 | Diarrhea | Moribund |

# Table S3

**Table S3.** Histopathological scores

| Score | Inflammation | Epithelial hyperplasia | Erosion and ulceration | Extent of lesion |
|---|---|---|---|---|
| 0 | absent | None | None | 0 |
| 1 | minimal in mucosa | mild | rare erosions | <10 |
| 2 | mild affecting mucosa and sub-mucosa | mild with minimum goblet cell loss | some erosions | 10-25 |
| 3 | moderate affecting mucosa, sub-mucosa, and sometimes transmural | moderate with mild goblet cell loss | multiple erosions and ulcerations with cryptitis and crypt abscesses | 25-50 |
| 4 | severe: often transmural | marked with moderate to marked goblet cell loss | ulcers associated with necrosis and fibrosis | >50 |

# Table S4

**Table S4.** Primer sequences

| Gene | Primer sequence |
|---|---|
| *rpoA*-F | ATTCGTCGTGCGGCTACCATTC |
| *rpoA*-R | GAGCGGACAGTCAATTCCAGATCG |
| *Tir*-F | CTTCAGGAATGGGAGATGGA |
| *Tir*-R | CAACCGCCTGAACAATACCT |
| *EspA*-F | AGTGATCTTGCGGCTGAGTT |
| *EspA*-R | ATCCACCGTCGTTGTCAAAT |
| *Map*-F | AGCGGTTGAAAGCGTGATAC |
| *Map*-R | CTTTACCGCACTGCTCATCA |
